# Notch signaling in serous ovarian cancer

**DOI:** 10.1186/s13048-014-0095-1

**Published:** 2014-11-04

**Authors:** Jolijn W Groeneweg, Rosemary Foster, Whitfield B Growdon, René HM Verheijen, Bo R Rueda

**Affiliations:** Vincent Center for Reproductive Biology, Department of Obstetrics and Gynecology, Massachusetts General Hospital, Boston, MA USA; Harvard Medical School, Boston, MA USA; Division of Gynecologic Oncology, Department of Obstetrics and Gynecology, Massachusetts General Hospital, Boston, MA USA; Division of Woman and Baby, Department of Gynecologic Oncology, University Medical Center Utrecht, Utrecht, The Netherlands

**Keywords:** Ovarian serous carcinoma, Notch, Gamma secretase inhibitor, Patient derived xenograft models

## Abstract

Ovarian cancer is the most lethal of all gynecologic malignancies because women commonly present with advanced stage disease and develop chemotherapy refractory tumors. While cytoreductive surgery followed by platinum based chemotherapy are initially effective, ovarian tumors have a high propensity to recur highlighting the distinct need for novel therapeutics to improve outcomes for affected women. The Notch signaling pathway plays an established role in embryologic development and deregulation of this signaling cascade has been linked to many cancers. Recent genomic profiling of serous ovarian carcinoma revealed that Notch pathway alterations are among the most prevalent detected genomic changes. A growing body of scientific literature has confirmed heightened Notch signaling activity in ovarian carcinoma, and has utilized *in vitro* and *in vivo* models to suggest that targeting this pathway with gamma secretase inhibitors (GSIs) leads to anti-tumor effects. While it is currently unknown if Notch pathway inhibition can offer clinical benefit to women with ovarian cancer, several GSIs are currently in phase I and II trials across many disease sites including ovary. This review will provide background on Notch pathway function and will focus on the pre-clinical literature that links altered Notch signaling to ovarian cancer progression.

## Background

Ovarian cancer represents the most lethal gynecologic malignancy in the United States. In 2014 alone, approximately 22,000 women are estimated to be diagnosed with ovarian cancer and more than 14,000 deaths attributed to the disease are projected to occur [1]. This high mortality is explained in part by the advanced disease stage at the time of diagnosis with approximately 75% of the patients presenting with stage III - IV disease [2]. Therapeutic strategies include cytoreductive surgery followed by six cycles of platinum and taxane based chemotherapy [3,4]. Despite the fact that the majority of patients achieve complete clinical remission following this treatment regimen, the prognosis of ovarian cancer remains unfavorable with a 5-year survival rate of approximately 50% [4]. This poor outcome is mainly attributed to the development of recurrent disease that is often resistant to chemotherapy [5,6]. Treatment options for recurrent ovarian cancer are currently limited and not curative, warranting the development of novel therapeutic strategies.

Epithelial ovarian malignancies comprise heterogeneous tumors, both on a cellular and a molecular level. A recently developed dualistic model divides the different ovarian cancer subtypes into type I and type II carcinomas [7]. Type I tumors comprise low-grade serous, low-grade endometrioid, clear cell and mucinous ovarian cancers. They are characterized by indolent, genetically stable tumors that arise from a precursor lesion, often present at an early stage and are relatively resistant to cytotoxic therapy. In contrast, the more prevalent type II tumors are highly aggressive, present at an advanced stage, typically harbor *TP53* mutations that lead to genetic instability, and are initially more sensitive to chemotherapeutic agents. While high-grade serous carcinomas account for the vast majority of type II ovarian cancers, other subtypes include high-grade endometrioid ovarian carcinoma and carcinosarcomas [8,9]. In recent years, multiple genetic and epigenetic abnormalities as well as changes in molecular pathways have been identified that are often characteristic for specific histologic subtypes [10,11]. Therapeutic targeting of the molecular aberrations and cellular signaling pathways involved in tumor progression may provide novel treatment options for women with recurrent ovarian cancer. This review will focus on the role of the Notch signaling cascade in high-grade serous ovarian cancer and the potential therapeutic effectiveness of Notch pathway inhibition in this disease.

### The Notch signaling pathway

#### Functions of Notch signaling

The evolutionary conserved Notch pathway was first discovered in Drosophila a century ago, when flies with a mutation in the Notch gene were found to have wing deformities [12]. The functional significance of the Notch signaling cascade has been well established in neural development [13,14] and has since been established in multiple cellular processes, during embryonic development and in self-renewing adult tissues [15,16]. The Notch pathway functions through cell-to-cell contact and is involved in the regulation of proliferation, differentiation and apoptosis, depending on the cellular context [17,18]. In adult tissues, Notch signaling acts to control tissue homeostasis and stem cell maintenance.

#### Notch receptors and ligands

**Figure 1:**
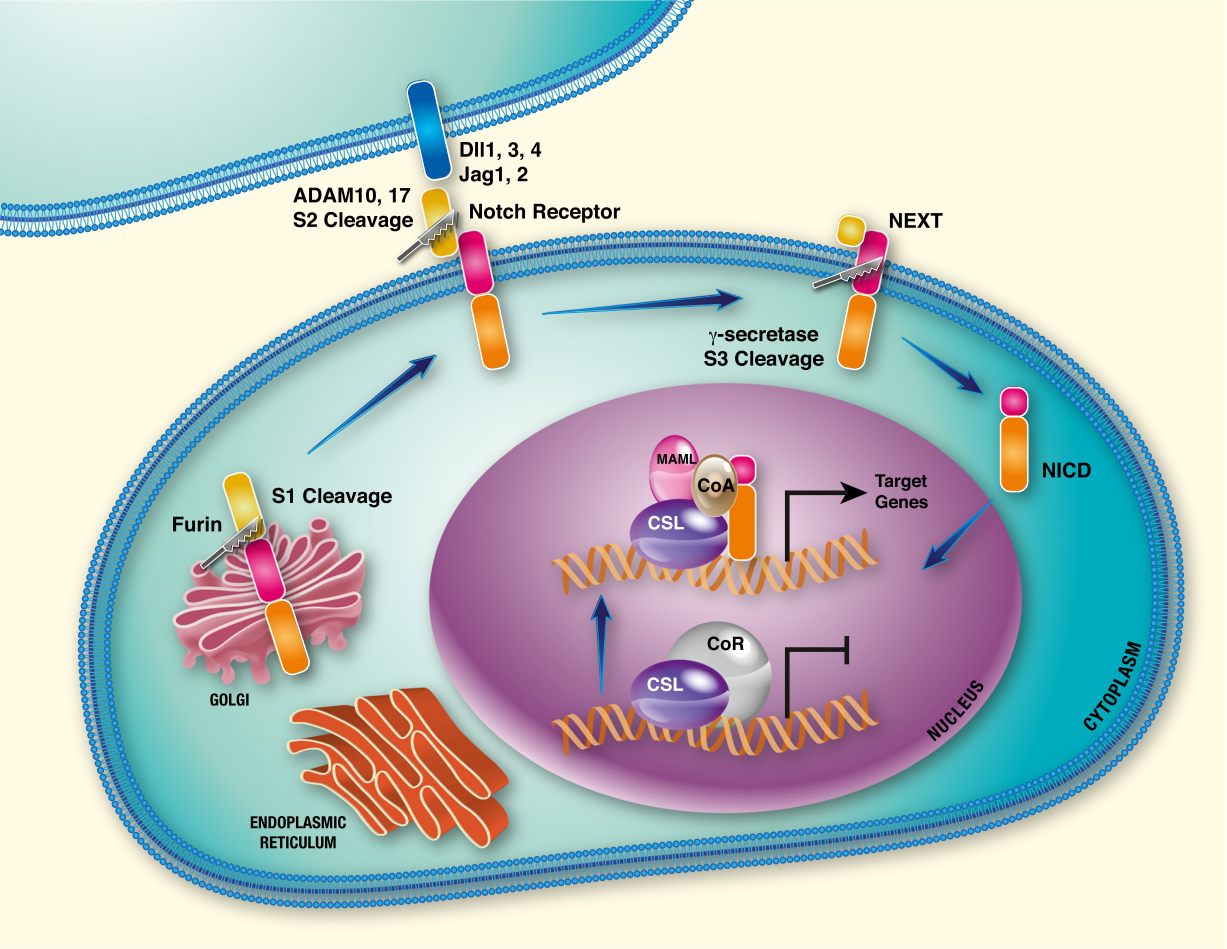
**The Notch signaling cascade is activated by cell-cell interaction.**

Thus far, four Notch receptors (Notch1-4) and five ligands have been identified in mammals. Three ligands belong to the Delta-like family (Dll1, 3 and 4) and two ligands (Jagged1 (Jag1) and Jagged2 (Jag2)) are Serrate-like [19-23]. Notch receptors as well as their ligands are single-pass transmembrane proteins with extracellular domains that consist of multiple epidermal growth factor (EGF)-like repeats [24,25]. The receptors are synthesized as inactive precursors in the endoplasmic reticulum that are proteolytically cleaved by furin-like convertases in the trans-Golgi compartment [26]. This first cleavage, termed S1, results in an extracellular N-terminal fragment and a transmembrane C-terminal fragment that also includes the Notch intracellular domain (NICD). Finally, non-covalent binding between the two fragments forms the mature Notch heterodimeric receptor [27] (Figure 1). During the process of Notch receptor synthesis, the extracellular fragment is glycosylated by Fringe glycosyltransferases, which modifies the binding affinity between the receptor and its ligands [28,29].

#### Signaling cascade

As shown in Figure 1, Notch signaling is activated by a receptor-ligand binding between two neighboring cells, leading to a conformational change of the Notch receptor and exposure of a cleavage site (S2) in its extracellular domain [30,31]. S2 cleavage by A Disintegrin And Metalloprotease (ADAM)10 or 17 produces an intermediate transmembrane fragment termed NEXT (Notch extracellular truncation) which is accessible to gamma-secretase for S3 cleavage [32]. The gamma-secretase complex consists of four subunits: the catalytic subunit presenilin, nicastrin, APH-1 and PEN-2 [33]. S3 cleavage by gamma-secretase leads to release of the NICD, which translocates to the nucleus and binds to the DNA bound CBF-1/Su(H)/Lag-1 protein complex (CSL, also known as RBP-jκ) that constitutively represses transcription in the absence of NICD [34,35]. The NICD displaces a co-repressor complex from CSL and recruits co-activators such as Mastermind-like 1 (MAML1), allowing the transcription of Notch target genes [34,36].

#### Notch target genes

The most well-known Notch target genes are transcription factors of the Hairy/Enhancer of Split (*Hes*) and Hes-related (*Hey*) families [37]. Hes and Hey members are helix-loop-helix proteins, forming homo-or heterodimers that regulate transcription of genes involved in cell fate determination [37-39]. Other Notch pathway targets include cell cycle regulators cyclin D1 and p21, NF-κB family members, c-Myc and Deltex [40-43].

#### Crosstalk with other signaling pathways

The Notch pathway is part of a complex network of developmental signaling pathways that also includes the Hedgehog, Wnt, receptor tyrosine kinases (RTK), transforming growth factor-β (TGF-β) and Janus kinase/signal transducers and activators of transcription (Jak/STAT) pathways [44,45]. The Notch pathway has also been implicated as interacting with the EGFR/HER2 receptor tyrosine kinase family, as well as phosphatidylinositol 3-kinase/AKT/mTOR signaling cascade, two central growth pathways in the physiologic and neoplastic setting [46]. Although integration of Notch signaling with signals from other pathways has been a focus of investigation, the exact mechanisms of this crosstalk remain largely unknown. The interaction of Notch signaling with other pathways highly depends on the cellular context, and differs from one cellular environment to another. For example, the Notch and Wnt pathways were found to cooperate in maintaining undifferentiated hematopoietic stem cells [47], whereas an antagonistic interaction between Wnt and Notch has been reported in Drosophila development [48]. Similarly, opposed functions in distinct cellular contexts have been reported for the Notch and Ras pathways. Notch signaling was shown to complement the Ras pathway during Drosophila eye development [49], while inhibition of the Ras pathway by Notch signaling has been demonstrated in *C. elegans* hermaphrodite vulval development [50,51]. Activation of Sonic Hedgehog (Shh) led to upregulation of Notch signaling and determination of arterial cell fate in zebrafish [52], and induction of Shh has been observed in murine somatic and human embryonic stem cells following Notch receptor activation [53]. The phosphatidylinositol 3-kinase (PI3K)-AKT and Notch signaling cascades were found to interact agonistically during murine hematopoietic stem cell differentiation and in a variety of human cell types including T-cells and neurons [54,55]. Negative crosstalk between the EGFR and Notch pathways has been described in Drosophila wing development [56,57], while Notch signaling was shown to activate Her2 (ERBB2) in human embryonic kidney cells [58].

#### Notch signaling in cancer

Oncogenic Notch signaling was first described in T-cell acute lymphoblastic leukemia (T-ALL), in which a chromosomal translocation event generates a constitutively active variant of Notch1 [59]. Later studies have shown that Notch1 activating mutations occur in the majority of T-ALLs [60]. Deregulation of Notch receptors, ligands or targets has since been described in a variety of solid and hematological tumors including breast [61,62] pancreatic [63], brain [64], lung [65], ovarian [11], head and neck [66] and colorectal [67] cancer, as well as leukemia [60,68], lymphomas [69,70] and multiple myeloma [71]. While these and other studies demonstrate Notch pathway involvement in tumor initiation and progression, tumor suppressive roles of Notch signaling have also been reported [72,73]. Most notably, Notch functions as a tumor suppressor in the skin, and loss-of-function mutations in Notch receptors have been identified in cutaneous squamous cell carcinoma [74,75]. Although little is known about the mechanisms behind these contradictory actions of the Notch pathway in cancer, it is generally assumed that the various outcomes of Notch signaling depend on interactions with the microenvironment and crosstalk with other signaling pathways [72].

#### Notch signaling in serous ovarian cancer

In ovarian cancer, a role for Notch signaling was first discovered in two studies aimed at identifying potential diagnostic markers of epithelial ovarian cancer. Gene expression of human ovarian cancer samples or cell lines was evaluated and compared to normal ovarian surface epithelium samples or cell lines, respectively [76,77]. One study reported upregulation of the *Notch3* gene in all analyzed ovarian cancers [76], while the second study reported increased *Jag2* gene expression in ovarian cancer cell lines compared with benign controls [77]. Subsequently, Park et al. used a single nucleotide polymorphism (SNP) array to analyze DNA copy number alterations in high-grade serous ovarian carcinomas and identified an amplicon corresponding to the *Notch3* locus in 20% of cases. *Notch3* gene amplification correlated with Notch3 protein overexpression, as determined by fluorescent *in situ* hybridization and immunohistochemistry (IHC) [78]. These results were confirmed by others, who demonstrated amplification of the *Notch3* gene using a SNP array in 21% of analyzed ovarian cancers [79]. Moreover, recent large-scale genomic and epigenomic analyses of high-grade serous ovarian carcinomas by The Cancer Genome Atlas (TCGA) Network revealed altered Notch signaling in 22% of cases with alterations in *Notch3* occurring in 50% of those cases [11]. In a study of Notch ligands, Jag1 was shown to be the most highly expressed ligand in both ovarian cancer cells and surrounding peritoneal mesothelial cells with interaction of Jag1 and Notch3 resulting in activation of the signaling cascade and promotion of cell proliferation and adhesion [80]. In addition, *Pbx1* and *DLGAP5* have been identified as direct target genes of Notch3 in ovarian cancer, and knockdown of either target with shRNAs led to reduced cell proliferation and, in the case of *Pbx1* knockdown, impaired tumor formation [81,82]. More recently, the TCGA data generated from 488 ovarian cancer patients were used to analyze epigenetic changes of genes of the Notch superfamily. Inverse correlations were found between DNA methylation and expression of the Notch pathway target genes *CCND1* and *PPARG* and the Notch-interacting gene *RUNX1*. Additionally, an inverse correlation in expression was established between *CCND1*, *PPARG* and *RUNX1* and specific miRNAs that regulate each of those genes. Subsequent survival analyses revealed significantly poorer overall survival rates of patients with high *CCND1*, *PPARG* and *RUNX1* gene expression and low methylation or low levels of the relevant miRNAs compared with patients with low gene expression and high methylation or high miRNA levels [83]. Furthermore, several groups have linked Notch3 expression to the clinical prognosis of ovarian cancer. Using quantitative reverse transcription polymerase chain reaction (RT-PCR) and IHC, Jung *et al.* observed elevated mRNA levels of *Notch3*, *Jag1* and *Jag2* as well as higher Notch3 and Jag2 protein expression in serous ovarian cancer samples as compared to benign controls. High *Notch3* mRNA expression correlated significantly with worse overall survival and clinical chemoresistance, and Notch3 protein overexpression was significantly associated with the prognostic parameters advanced stage disease, lymph node metastases and distant metastases [84]. Moreover, elevated nuclear Notch3 immunostaining has been found in recurrent serous ovarian carcinoma specimens as compared to primary ovarian cancer samples from the same patients. A significant association between high *Notch3* mRNA or nuclear Notch3 protein levels and worse overall and progression-free survival rates was also described [85,86]. Ectopic expression of Notch3 following transduction with a Notch3 intracellular domain (NICD3) in ovarian surface epithelium and low-grade serous ovarian cancer cell lines with low endogenous Notch3 expression led to increased resistance to carboplatin *in vitro*. Inversely, shRNA-mediated knockdown of Notch3 in OVCAR3 cells resulted in higher sensitivity to carboplatin, compared with OVCAR3 cells transduced with a non-specific control shRNA [85].

The role of Notch1 in ovarian cancer was first studied by Hopfer et al., [87] who evaluated mRNA expression of Notch pathway members in ovarian adenocarcinomas, borderline tumors and adenomas and demonstrated more frequent expression of *Jag2* and *DLL1* in adenocarcinomas as compared to adenomas. Although quantitative RT-PCR and Western blot analyses revealed similar Notch1 expression levels in ovarian adenocarcinomas and adenomas, stable transfection of A2780 ovarian cancer cells with the intracellular domain of Notch1 (NICD1) increased cell proliferation and enhanced colony-formation capacity, suggesting a role for Notch1 signaling in ovarian tumor growth [87]. Analyses of the NICD1 protein by other investigators revealed high NICD1 levels in OVCAR3, SKOV3 and CaOV3 cell lines. Additionally, NICD1 was expressed in 76% of primary human serous ovarian cancer samples, as assessed by Western blotting. Subsequent siRNA downregulation of NICD1 in all three ovarian cancer cell lines resulted in inhibition of cell proliferation [88]. Results obtained from PCR, immunoblotting or functional studies of Notch1 have been easily interpreted. In contrast, IHC analyses of Notch1 expression in ovarian cancer have produced variable results. Wang and colleagues used IHC to study Notch1 levels in ovarian cancer specimens of various histological grades as well as patient-matched contralateral benign ovarian samples and normal ovarian tissues. Notch1 immunostaining was observed in 95% of analyzed serous ovarian cancer specimens versus 8% and 6% of matched benign controls and normal ovarian samples, respectively. Notch1 immunostaining was predominantly found in the cell membrane and cytoplasm. Positivity scores correlated with histological grade and clinical disease stage, and the IHC findings were confirmed by Western blotting and quantitative RT-PCR [89]. An IHC protocol was recently developed to detect the gamma-secretase cleaved NICD1. In contrast to the findings of the full-length Notch1 IHC studies, NICD1 was not expressed in any of the 147 analyzed ovarian cancer specimens although a subset of samples from other cancer types showed nuclear NICD1 immunostaining with this method [90]. In another study, IHC analysis of 10 serous ovarian tumors by others detected expression of Notch1, Jag1 and Dll1 in both cytoplasm and nucleus, and the observed Notch1 protein levels correlated significantly with metastasis in this small cohort [91].

#### Notch signaling in serous ovarian cancer stem cells

A few investigations have shown elevated Notch levels in recurrent or chemoresistant ovarian cancer. In ovarian cancer and many other malignancies, the development of recurrent disease has been attributed, at least in part, to a tumorigenic and chemotherapy resistant sub-population of cancer cells called tumor-initiating or cancer stem cells (CSCs) [92-94]. Consistent with this hypothesis, increased expression of CSC markers and enrichment of the side population (cells showing increased efflux of Hoechst dye as identified by flow cytometry, reviewed in Foster et al., [95]) have been observed in ovarian cancer samples following platinum based chemotherapy, compared with chemotherapy naive cells [96,97]. Similarly, increased resistance to cisplatin and paclitaxel has been demonstrated in sphere forming primary serous ovarian cancer cells, cultured under stem cell-selective conditions, compared with the same cells cultured under differentiating conditions. These spheroid ovarian CSCs also showed elevated levels of the CSC marker proteins CD44 and CD117 as well as an increase in mRNA levels of *Notch1* and other stem cell genes, compared with differentiated cells and parental bulk tumor cells [98]. Retrovirus-mediated overexpression of the Notch3 intracellular domain (NICD3) in ovarian surface epithelium and low-grade serous ovarian cancer cell lines with low endogenous Notch3 levels led to upregulation of the stem cell associated genes *Nanog* and *Oct4*, further suggesting a role of the Notch pathway in CSC function [85]. Furthermore, gene microarray analyses of serous ovarian cancer side population cells from ascites samples showed upregulation of three genes involved in Notch signaling, compared with main population cells. These genes, *ADAM19*, *FPTG* and *ST3GAL6*, were also found to be overexpressed in recurrent ovarian cancer specimens, compared with matched primary samples [99]. Supporting these findings, Steg and colleagues have reported increased transcript levels of stem cell pathway genes, including the Notch pathway member presenilin 2 (*PSEN2*), in recurrent ovarian cancer samples compared with matched primary tumors [96]. Collectively, these studies provide evidence suggesting that Notch signaling is enhanced in ovarian CSCs, as compared to tumor bulk cells.

#### Notch signaling and epithelial-to-mesenchymal transition in serous ovarian cancer

In addition to its role in CSCs, the Notch pathway has been implicated in epithelial-to-mesenchymal transition (EMT) and may thereby promote tumor invasiveness and metastasis [100]. The process of EMT has been associated with chemoresistance and stem cell-like characteristics in several cancers [101], including ovarian cancer [102-104]. A recent study has demonstrated that overexpression of NICD3 in OVCA429 serous ovarian cancer cells induces EMT, as confirmed by a fibroblast-like cell morphology as well as upregulation of the mesenchymal markers Slug, Snail and smooth muscle α-actin and down regulation of the epithelial marker E-cadherin. The OVCA429/NICD3 cells showed increased resistance to carboplatin-induced apoptosis compared with OVCA429 control cells [105]. Although further investigation of the role of the Notch pathway in ovarian EMT is needed, these findings suggest that activation of Notch can induce EMT in serous ovarian carcinomas.

#### Notch signaling and angiogenesis in serous ovarian cancer

While Notch signaling has been linked extensively to tumor angiogenesis [106-108] in many malignancies, few studies have described a similar role of the pathway in ovarian tumor angiogenesis [109,110]. Lu and colleagues investigated gene expression differences between endothelial cells from high-grade serous ovarian carcinomas and endothelial cells from benign ovaries using gene microarrays. Overexpression of 23 genes was found in ovarian tumor endothelial cells as compared to benign ovarian endothelial cells. Among these upregulated genes was *Jagged1*, and silencing of this gene with a siRNA decreased tube formation and migration of endothelial cells [109]. Other investigators observed Dll4 overexpression in tumor and endothelium in 72% of ovarian cancer samples analyzed by IHC, and increased Dll4 levels were associated with worse overall survival when compared to samples with low Dll4 expression. When *DLL4* was silenced *in vivo* using nanoparticle delivery of a *DLL4* specific siRNA to mice harboring A2780 or SKOV3ip1 cell line derived xenografts, the targeting of both tumor cells and tumor-associated mouse endothelial cells inhibited tumor growth and deregulated angiogenesis. Moreover, tumor growth was further inhibited when the vascular endothelial growth factor inhibitor bevacizumab was added to this treatment regimen [110] suggesting synergy between anti-VEGF treatment and Dll4 targeting. In summary, the described studies provide evidence to suggest that the Notch pathway is not only involved in epithelial ovarian tumor growth, but also plays a role in endothelial cell function and angiogenesis of serous ovarian tumors.

#### Therapeutic targeting of the Notch pathway in serous ovarian cancer

The growing body of evidence regarding the role of Notch signaling in cancer has led to the development of different Notch pathway inhibitors, a number of which are in current clinical trials (see Table 1). Several steps in the pathway can be targeted, and established classes of inhibitors include monoclonal antibodies against Notch ligands or receptors, receptor decoys, gamma-secretase inhibitors (GSIs), peptides that block the nuclear transcriptional complex, and natural compounds [111,112]. GSIs are the most widely studied Notch pathway targeting agents. A variety of GSIs with distinct chemical structures but similar biological activity have been developed and each [113] inhibits signaling through all four Notch paralogs by preventing formation of the active NICD. Pre-clinical studies in a variety of cancers have shown inhibition of tumor growth or cell proliferation by GSIs [114-118]. In early phase clinical trials of GSIs, promising anti-tumor effects in several solid malignancies have been observed [119-122] despite dose-limiting toxicities evidenced mainly by gastrointestinal events. Over 40 clinical trials investigating the efficacy of GSI therapy in solid or hematologic cancers are ongoing or have recently been completed (see Table 1 for current list of ongoing trials). A subset of these trials use GSI in combination with standard chemotherapy or other targeting agents (([112]) https://clinicaltrials.gov/).

**Table 1:** **Ongoing phase I and phase II trials of therapies targeting the Notch pathway**

**Drug**	**Target**	**ClinicalTrials.gov identifier**	***n***	**Delivery**	**Disease site**	**Monotherapy or combination**
MK-0752	γ-secretase	NCT01098344	60	Oral	Unresectable pancreatic cancer	Combination with gemcitabine
RO4929097	γ-secretase	NCT01071564	46	Oral	Unresectable breast cancer	Combination with vismodegib
RO4929097	γ-secretase	NCT01238133	14	Oral	Neoadjuvant breast cancer	Combination with carboplatin and paclitaxel
RO4929097	γ-secretase	NCT01122901	60	Oral	Glioblastoma	Monotherapy
RO4929097	γ-secretase	NCT01151449	30	Oral	Breast cancer	Monotherapy
		NCT01120275	24		Melanoma	
		NCT01232829	21		Pancraetic cancer	
RO4929097	γ-secretase	NCT01119599	34	Oral	Glioblastoma	Combination with radiation therapy and temozolomide
RO4929097	γ-secretase	NCT01193881	39	Oral	Lung cancer	Combination with erlotinib
RO4929097	γ-secretase	NCT01158274	30	Oral	Refractory solid tumors	Combination with capecitabine
RO4929097	γ-secretase	NCT01141569	5	Oral	Renal cell cancer	Monotherapy
RO4929097	γ-secretase	NCT01189240	13	Oral	Glioblastoma	Combination with bevacizumab
RO4929097	γ-secretase	NCT01200810	78	Oral	Refractory prostate cancer	Combination with bicalutamide
RO4929097	γ-secretase	NCT01175343	37	Oral	Refractory ovarian, fallopian tube or peritoneal cancer	Monotherapy
BMS-906024	γ-secretase	NCT01653470	95	IV	Advanced/metastatic solid tumors	Combination with weekly paclitaxel; 5-FU and irinotecan; carboplatin and paclitaxel
BMS-906024	γ-secretase	NCT01292655	110	IV	Advanced/metastatic solid tumors	Monotherapy
BMS-906024	γ-secretase	NCT01363817	42	IV	T-cell leukemia or lymphoma	Monotherapy
BMS-986115	γ-secretase	NCT01986218	40	Oral	Advanced/metastatic solid tumors	Monotherapy
PF-03084014	γ-secretase	NCT01981551	17	Oral	Desmoid tumors	Monoterapy

#### Preclinical studies of Notch inhibition with GSI in serous ovarian cancer*: in vitro* models

Several groups have studied the *in vitro* effectiveness of GSIs in serous ovarian cancer. A reduction of cell proliferation and induction of apoptosis have been reported in OVCAR3 and A2780 ovarian cancer cell lines following administration of the compound GSI-1, compared with DMSO controls [78]. The same GSI was used by others who showed decreased cell proliferation post-treatment in A2780 and cisplatin resistant KFr13 serous ovarian cancer cell lines which both express high levels of NICD3 [86]. Moreover, it was shown that treatment of A2780 cells with the GSI DAPT decreased cell proliferation in a dose- and time-dependent manner, inhibited colony formation and induced cell cycle arrest and apoptosis while reducing Notch1 and Hes1 mRNA and protein levels [123]. In contrast with these findings, a recent study showed unchanged proliferation rates of OVCAR3, SKOV3 and several other ovarian cancer cell lines following treatment with the GSIs Compound E, DAPT or dibenzazepine (DBZ) [94].

#### Preclinical studies of Notch inhibition with GSI in serous ovarian cancer*: in vivo* models

Limited studies have investigated Notch inhibition *in vivo* for the treatment of ovarian cancer. Using *in vitro* and *in vivo* ovarian cancer models, McAuliffe and colleagues have investigated both the anti-tumor efficacy of GSI-1 as a single agent and in combination with cisplatin, and the effects of these treatments on ovarian CSC sub-populations. These investigators observed a reduction in cell viability following GSI treatment and a synergistic response to combined GSI and cisplatin therapy in established cell lines and cultured cells derived from ascites samples of platinum resistant and platinum sensitive patients. Sensitivity to GSI was found to correlate with Notch3 protein levels, as assessed by Western blotting. siRNA-mediated knockdown of Notch3 increased sensitivity to cisplatin in PA-1 and OVCAR3 cell lines and had no effect in SKOV3 cells which do not express detectable levels of Notch3. The described synergy of GSI and cisplatin was confirmed *in vivo*, using xenografts derived from these established cell lines. In addition, *in vitro* treatment with GSI or GSI + cisplatin decreased the side population and *in vivo* GSI therapy reduced both the side population and mRNA levels of the CSC markers *CD44* and *ALDH1*, suggesting GSI is effective at eliminating CSCs [124]. The *in vivo* effect of single agent GSI or GSI in combination with standard chemotherapy in a patient derived xenograft (PDX) model was explored by our group using cohorts of mice harboring PDX tumors derived from either clinically platinum sensitive or clinically platinum resistant primary ovarian serous tumors. Three of four platinum sensitive tumors and one of three platinum resistant tumors showed a decrease in tumor growth following single agent GSI treatment. Moreover, combination treatment with GSI and paclitaxel led to a markedly greater reduction in tumor growth compared with paclitaxel alone and GSI alone in all platinum resistant tumors. The combination of GSI with paclitaxel and carboplatin was not more effective than paclitaxel/carboplatin alone in platinum sensitive tumors [125]. These findings highlight a potential role for Notch pathway inhibition in addition to cytotoxic therapy in the recurrent, platinum resistant setting in serous ovarian cancer.

#### Clinical studies of Notch inhibition with GSI in serous ovarian cancer

To date, early clinical trials have provided little data regarding the efficacy of GSIs in ovarian cancer patients. A recent phase I clinical trial using the GSI RO4929097 in a range of advanced solid tumors reported prolonged stable disease in three of nine ovarian cancer patients [122]. In other phase I studies, no clinical benefit of RO4929097 in combination with gemcitabine was found in two of two ovarian cancer patients included in the trial [119], and the GSI MK-0752 was clinically ineffective as single agent therapy in three of three ovarian tumors [120]. Tables 1 and 2 highlight ongoing trials of various GSIs. While the only phase II trial exclusively in ovarian carcinoma assesses RO4929097 as a single agent, many other disease sites are testing GSI in combination with other biologics or cytotoxic chemotherapies. In the reported trial literature, the most clinical benefit has been observed in those trials that combine GSI with other agents suggesting a supporting therapeutic role for GSI. The fact that monotherapy has resulted in lower published clinical benefit rates stems in part from phase I trial designs that are powered to detect toxicity, not efficacy. However, an additional contributing factor is that durable tumor control from Notch inhibition requires careful selection for tumors innately dependent on Notch signaling for proliferation.

**Table 2:** **Completed phase I and phase II trials of therapies targeting Notch pathway**

**Drug**	**Target**	**ClinicalTrials.gov identifier**	***n***	**Delivery**	**Disease site**	**Monotherapy or combination**	**CR (%)**	**PR (%)**	**SD (%)**
MK-0752	γ-secretase	NCT00106145 [120]	103	Oral	Solid tumors	Monotherapy	1 (1)	0 (0)	10 (10)
MK-0752	γ-secretase	NCT00645333 [121]	30	Oral	Breast cancer	Combination with docetaxel	0 (0)	11 (42)	9 (34)
MK-0752	γ-secretase	NCT00572182 [126]	23	Oral	Refractory Pediatric CNS cancer	Monotherapy	0 (0)	0 (0)	2 (9)
RO4929097	γ-secretase	NCT01198184 [127]	17	Oral	Solid tumors	Combination with temsirolimus	0 (0)	0 (0)	11 (73)
RO4929097	γ-secretase	NCT01145456 [119]	18	Oral	Solid tumors	Combination with gemcitabine	0 (0)	1 (6)	4 (22)
RO4929097	γ-secretase	NCT01131234 [128]	20	Oral	Solid Tumors	Combination with cediranib	0 (0)	1 (5)	11 (55)
RO4929097	γ-secretase	NCT01116687 [129]	33	Oral	Metastatic colorectal cancer	Monotherapy	0 (0)	0 (0)	6 (18)
RO4929097	γ-secretase	NCT01232829 [130]	12	Oral	Refractory pancreatic cancer	Monotherapy	0 (0)	0 (0)	3 (25)

To date, no trials testing Notch pathway inhibition in patients pre-selected based on alterations in the Notch pathway have been reported. Significant pre-clinical data, however, seem to support such stratification as a promising approach. Future clinical studies will need to employ clinical as well as scientific endpoints to understand better the molecular characteristics of tumors that respond to therapy. Without a clear biomarker, specific biologic therapies such as GSI, are unlikely to become a mainstay of therapy. In addition to biomarker discovery, more trials investigating combination of GSI with conventional cytotoxics, such as taxanes needs to be conducted. Pre-clinical rationale exists for this combination approach as tumor cell resistance to cytotoxics and other biologic therapies, such as taxanes and anti-HER2 therapies, has been linked to heightened Notch signaling [131,132]. Since Notch may be an important escape pathway that reconstitutes the oncogenic potential of a tumor cell, Notch inhibition may be a key adjunct to conventional therapies known to be effective [133].

#### Other Notch inhibitors in serous ovarian cancer

In addition to targeting of gamma-secretase activity, alternative methods of Notch pathway inhibition have been studied in ovarian cancer. Inhibition of Jag1 using siRNA constructs in the IGROV-AF1 cell line and the taxane resistant SKOV3Trip2 cell lines resulted in a reduction of cell viability as well as sensitization to docetaxel in SKOV3Trip2 cells. The same cell lines were used to generate intraperitoneal tumors in mice, and the effects of targeting Jag1 in stromal and malignant cells were evaluated by treatment of mice with either mouse specific *Jag1* siRNA, human specific *Jag1* siRNA or both siRNA constructs. Either siRNA alone reduced tumor growth, and the combination of mouse and human *Jag1* siRNAs showed a synergistic effect in tumors derived from both cell lines. Combination treatment of SKOV3Trip2-derived tumors with human and mouse specific *Jag1* siRNAs and docetaxel led to the highest decrease in tumor weight, compared with the siRNAs alone or docetaxel alone. Microvessel densities were reduced after treatment with anti-murine *Jag1* siRNA, suggesting this therapy induced anti-angiogenic effects [134]. Finally, the natural compounds xanthohumol and withaferin A inhibited cell growth and induced apoptosis and cell cycle arrest in ovarian cancer cell lines through downregulation of Notch1 (withaferin A and xanthohumol) and Notch3 (withaferin A) [135,136].

## Conclusions

Considerable evidence supports an important oncogenic role of Notch signaling in high-grade serous ovarian cancer. Perturbation in normal regulation of Notch1 and Notch3 as well as Notch ligands, target genes and other members of the Notch pathway has been described. The many biological and clinical aspects of ovarian tumorigenesis in which aberrant Notch signaling appears to be involved include tumor initiation and progression, metastasis, resistance to chemotherapy, CSC activity, angiogenesis and EMT. Despite its functional complexity and crosstalk with other signaling cascades, the Notch pathway represents an attractive therapeutic target in ovarian cancer. Pre-clinical analysis of Notch inhibition suggests Notch targeting agents such as GSIs hold promise as potential treatment strategies for ovarian cancer patients, most notably in the setting of recurrence and chemoresistance. In addition, combination regimens with conventional cytotoxic therapy as well as other targeted therapies warrant further investigation.
